# Biomimetic Deposition of Hydroxyapatite Layer on Titanium Alloys

**DOI:** 10.3390/mi12121447

**Published:** 2021-11-25

**Authors:** Madalina Simona Baltatu, Andrei Victor Sandu, Marcin Nabialek, Petrica Vizureanu, Gabriela Ciobanu

**Affiliations:** 1 Faculty of Materials Science and Engineering, Gheorghe Asachi Technical University of Iasi, 41 D. Mangeron Street, 700050 Iasi, Romania; simona.baltatu@tuiasi.ro (M.S.B.); sav@tuiasi.ro (A.V.S.); 2 Romanian Inventors Forum, 3 Sf. P. Movila St, 700089 Iasi, Romania; 3 Department of Physics, Częstochowa University of Technology, 42-200 Częstochowa, Poland; nmarcell@wp.pl; 4 Faculty of Chemical Engineering and Environmental Protection Cristofor Simionescu, Gheorghe Asachi Technical University of Iasi, 73 D. Mangeron Street, 700050 Iasi, Romania; gciobanu03@yahoo.co.uk

**Keywords:** titanium alloys, biomaterials, TiMoZrTa system, biomimetic deposition

## Abstract

Over the last decade, researchers have been concerned with improving metallic biomaterials with proper and suitable properties for the human body. Ti-based alloys are widely used in the medical field for their good mechanical properties, corrosion resistance and biocompatibility. The TiMoZrTa system (TMZT) evidenced adequate mechanical properties, was closer to the human bone, and had a good biocompatibility. In order to highlight the osseointegration of the implants, a layer of hydroxyapatite (HA) was deposited using a biomimetic method, which simulates the natural growth of the bone. The coatings were examined by scanning electron microscopy (SEM), X-ray diffraction (XRD), micro indentation tests and contact angle. The data obtained show that the layer deposited on TiMoZrTa (TMZT) support is hydroxyapatite. Modifying the surface of titanium alloys represents a viable solution for increasing the osseointegration of materials used as implants. The studied coatings demonstrate a positive potential for use as dental and orthopedic implants.

## 1. Introduction

The recent focus of research is on biocompatible materials, which are artificial products that have been imposed by the needs of people, affected by disease or accident, to improve health, leading to increased life expectancy [1]. The main issue is the way the body accepts these materials, or biocompatibility [2]. From here, the main disadvantages of implants are determined by the nature, mode of synthesis and different physical and chemical properties of the materials used in relation to living tissues, such as: the ability to change its structure and properties according to the demands it supports (mechanical loading for bone tissue or blood flow for blood vessels) or self-healing ability [3,4]. Biomedical engineering, including the development of artificial implants such as knee and hip stents, has become an important international activity with a significant social impact [5]. Research contributes significantly to the understanding of the effects of mechanical forces on human recovery and locomotion and joint functionality. The boundless utilization of titanium (Ti) compounds in the area of implantology is principally because of the expanded consumption, obstruction and more elevated levels of biocompatibility. However, not all Ti combinations meet the prerequisites for biomedical applications. This has prompted the improvement of another collection of Ti amalgams with biocompatible components such as Mo, Zr, Ta, Nb, and so on, which as of late, have been introduced to the market to overcome the toxicity associated with Ti-V/Al-based alloys currently in use. In addition, because the surface of the biomaterial plays a key role in the cellular response, the improvement of biological and tribological properties by surface modification is often necessary [6,7,8].

Hydroxyapatite (Ca_10_(PO_4_)_6_ OH)_2_) is the major inorganic constituent in bone mass, weighing approximately 69% [9]. The most important property of hydroxyapatite as a biomaterial is its excellent biocompatibility, which is manifested in making direct connections with living bone tissue [10]. It is known that biological hydroxyapatite is a calcium phosphate that is found in the mineral structure of bone and tooth—in dentin and tooth enamel, but also in the case of pathological calcifications such as kidney stones, stones of the salivary gland, dental stones, etc. [11]. Hydroxyapatite can crystallize as a fine salt depending on the Ca/P ratio, the formation temperature, the presence of water or impurities and, depending on the preparation medium, in a humid environment at relatively low temperatures [12]. Hydroxyapatite is not only bioactive, but also osteoconductive and non-toxic [13]. The bonds they make with the bone are of a physico-chemical nature; the bone cells interact with the hydroxyapatite forming ionic, hydrogen and van der Waals bonds [13].

The notion of osteoconductivity has different meanings, depending on the field in which it is used [14]. Clinically, this means bone growth from the host bone tissue to the implant. Due to this meaning, any material (not only calcium phosphates, but also polymers) can be osteoconductive, based on the ability to regenerate the bone itself [15]. Current studies aim to obtain new materials for implants, as well as their biological and mechanical interfaces. The modification of surfaces, through chemical processes or the attachment of biomolecules, represents an important option for improving biocompatibility [16]. The properties of hydroxyapatite, including bioactivity, biocompatibility, solubility, mechanical and adsorption properties, can be adapted to a wide range of applications by controlling particle composition, size and morphology [17]. Hydroxyapatite in various forms such as powder, porous blocks or pearls can be used to fill bone defects and free spaces in the bone. These occur when portions of the bone have been removed due to disease (bone cancer) or when bone elongations are required (in dental applications). For medical applications, hydroxyapatite deposits are also obtained on solid supports (metallic or polymeric).

The surface modification techniques are listed and divided according to the governing processes and their purposes [18]. The main purpose of changing the metal surface is to improve the compatibility with hard tissue or to accelerate bone formation [19]. The development of biomimetic ceramic materials is of great interest in tissue and prosthetic engineering [20,21,22]. The most interesting property of hydroxyapatite is its ability to interact with living bone tissue, forming strong bonds with the bone. It is frequently used for orthopedic, dental and maxillofacial applications, either as a coating material for metal implants or as a bone-filling material [23,24]. Other studies report that various hip prostheses and component parts, based on metals or metal alloys, especially titanium alloys coated with a layer of hydroxyapatite, had the role of facilitating bone development on the implant. There are many ways to deposit hydroxyapatite on titanium alloys. In the field of coating techniques, several methods of deposition are mentioned, such as atmospheric plasma spraying, physical and chemical vapour deposition (PVD and CVD processes) [8]. In addition, in order to increase the bonding strength between coating and alloy substrate, thermal spraying technology is considered to be an effective coating preparation method, such as hot dip method [25,26], chemical vapor deposition method [27,28], slurry method [29,30], etc.

The aim of the present study was to obtain a hydroxyapatite coating on the surface of original titanium alloys. The apatite layers are obtained biomimetically by immersing the solid support in biomimetic solutions that mimic the vital fluids of the human body, at body temperature (37 °C). This induces the formation of a uniform and stable coating that can be developed in terms of the shape, structure and chemistry of the biomaterial. These hydroxyapatite coatings were characterized and tested for structural and mechanical characterization. Modifying the surface of titanium-based alloys is a viable solution for increasing the osseointegration of materials used as implants. The present article deals with three original alloys (Ti15Mo7Zr5Ta, Ti15Mo7Zr10Ta and Ti15Mo7Zr15Ta) obtained by the authors, then coated with an HA layer by the biomimetic method. For the assessment of the samples we used microstructural analysis, XRD, indentations tests and contact angle.

## 2. Materials and Methods

### 2.1. Obtaining of Ti-Mo-Zr-Ta Substrate Alloys

Three alloys of titanium alloyed with molybdenum, zirconium and tantalum were used as substrate materials. The elaboration of substrate alloys was elaborated with an electric vacuum melting installation by melting the elements, followed by melting the alloy seven times, a necessary operation in order to refine and homogenize the alloys. The melting of the elements took place uniformly, resulting in alloys with a precise and homogeneous chemical composition. The load introduced in the installation, as well as the mass of the semi-finished products after solidification, were weighed and are presented in Table 1 (approximately 70 g per alloy).

As raw materials, we used high purity elements such as Ti (99% purity), Mo (99% purity), Zr (99% purity) and Ta (99% purity), supplied by Alfa Aesar (Thermo Fisher Scientific, Kandel, Germany). The compositions of the substrate alloys are presented in Table 2.

### 2.2. Biomimetic Method

The samples involved in the study had a dimension of 10 mm × 10 mm × 3 mm. All the samples were polished with SiC paper and then cleaned in distilled water in an ultrasonic bath. After that, the samples were cleaned again for 15 min in acetone, 10 min in ethanol and 5 min in deionized water. The prepared samples were involved in a biomimetic treatment using an SBF solution that mimics the human blood plasma (Table 3), at 37 °C. The SBF solution was prepared by dissolving: NaCl, NaHCO_3_, KCl, Na_2_HPO_4_, MgCl_2·_6H_2_O, CaCl_2_·2H_2_O, Na_2_SO_4_, HCl and (CH_2_OH)_3_CNH_2_ (tris(hydroxymethyl)aminomethan) in deionized water, under vigorous stirring.

### 2.3. Morphological and Structural Analysis

To highlight the composition proportions obtained between the pure elements and the morphological elements of the alloys, we used the (SEM)—Quanta 200 3D Dual Beam and Vega 2 LSH by Tescan, Brno, Czech Republic with X-ray energy dispersion, at 20 kV. The sample used for the determination of the composition’s morphological and constituent phases had the dimensions of 10 mm × 10 mm × 5 mm.

Metallographic analysis provides the information on the micrographic structure of the alloy, its nature, shape, dimensions and distribution mode. A Zeiss Axio Imager A1 microscope (Oberkochen, Germany) was used for optical analyses. For metallographic preparing the samples were prepared with abrasive discs with a granulation between 300 and 3000 MPi, they were then polished with alumina suspension (1–6 µm), cleaned with alcohol, and ultrasonically cleaned in ethyl alcohol for 10 min. The samples prepared for microstructural analysis were etched with (10 mL HF, 5 mL HNO_3_, 85 mL H_2_O) solution.

The surface state of the samples was investigated via electron microscopy (SEM; Tescan Vega II LSH, SE detector, 20 to 30 kV electron gun voltage, VegaTC software). In order to see the cross-sectional morphology and interfacial bonding properties of the coating, the samples were cold embedded in an embedding support using a clamp for fixing and a special Epoxy resin for cold embedding.

Phase determination and crystalline structure were performed by qualitative X-ray diffraction analysis using an Expert PRO MPD facility from Panalytical (Almelo, The Netherlands), with Cu—X-ray tube (Kα-1.54051° Å), 2 theta: 10–90°, step size: 0.13°, time/step: 51 s, and a scan speed of 0.065651°/s. Highscore Plus software was used for processing the obtained data.

### 2.4. Micro-Indentation Test

Mechanical tests were performed for determining the tribological properties of the coated alloys using the UMTR 2M-CTR produced by CETR—Schaefer (CETR, Campbell, CA, USA). The samples prepared at specific dimensions were analyzed through micro-scale indentation on coated samples. This device can precisely control and measure the loads applied, displacements and velocities of the indenter. The hard indenters used in these tests were diamonds of conical shape with tip angles of 60°, moving with a constant force of 5 N, over a distance of 4 mm.

### 2.5. Contact Angle Test

Contact angles are measured by matching a mathematical expression to the droplet shape and then calculating the slope tangent to the droplet at the liquid-solid-vapor (LSV) interface line. The measuring material is introduced into the goniometer, and with a syringe, a single drop of water is applied on top of the test specimen. The goniometer is adjusted to measure the specific contact angle between the material and the edge of the drop.

For measuring the contact angle of titanium alloys covered with hydroxyapatite by the biomimetic method, a Kyowa DM-CE 1 goniometer (Takasaki, Japan) was used. The tests are designed to provide an accelerated method of determining the strength of the surface coating and also to test the performance of the cleaning mixture. The equipment ensures precisely controlled conditions, repeatability of results and close comparability of performance.

The contact angle is an extremely important metric indicator for understanding the surface properties of the material—adhesion, hygroscopicity and free energy of the solid surface. The contact angle is used to measure cleanliness, surface treatment effects, adhesiveness, and impermeability. Surfaces where the coating is tactile will have a high contact angle.

## 3. Results

### 3.1. Microstructural Analysis

**Figure 1:**
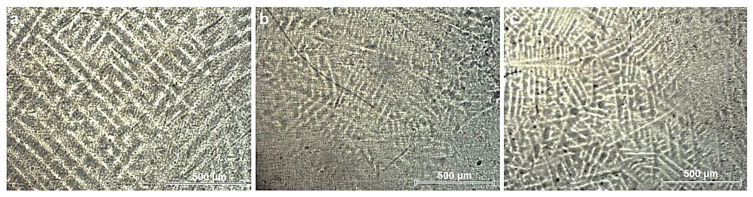
Microscopy images of substrate materials: (**a**) Ti15Mo7Zr5Ta; (**b**) Ti15Mo7Zr10Ta; (**c**) Ti15Mo7Zr15Ta.

Figure 1 illustrates the microstructural analysis of the substrate alloys. Alloys present coarse grained, lamellar microstructures with large β-grains and a lamellar matrix of alternating α and β. This type of lamellar structure is specific to titanium alloys depending on the percentage of beta stabilizing elements. All alloys contain a large amount of beta stabilizing elements (Mo, Ta) that lead to this type of structure.

**Figure 2:**
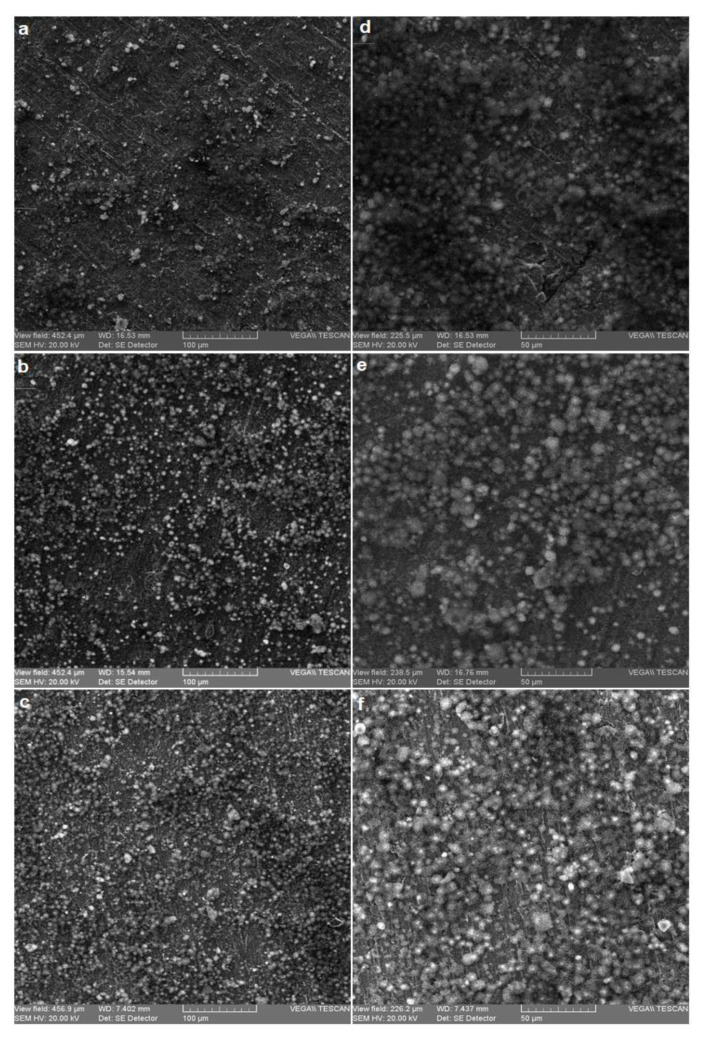
Structural aspects of the surface of coated samples at 500×: (**a**) S1-HA; (**b**) S2-HA; (**c**) S3-HA; and 1000×: (**d**) S1-HA; (**e**) S2-HA; (**f**) S3-HA.

Morphological aspects of coated samples with hydroxyapatite coating are presented in Figure 2.

The images confirmed that the hydroxyapatite coatings on the titanium surface were obtained, and the coatings were uniform.

After modifying the surface, the latest research suggests that the application of various biomimetic coatings can be beneficial for implant therapy success [31,32,33,34].

**Figure 3:**
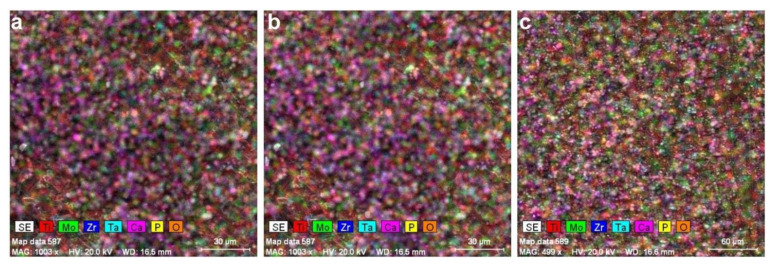
EDS elemental mapping of the metallic surface: (**a**) S1-HA; (**b**) S2-HA; (**c**) S3-HA.

**Figure 4:**
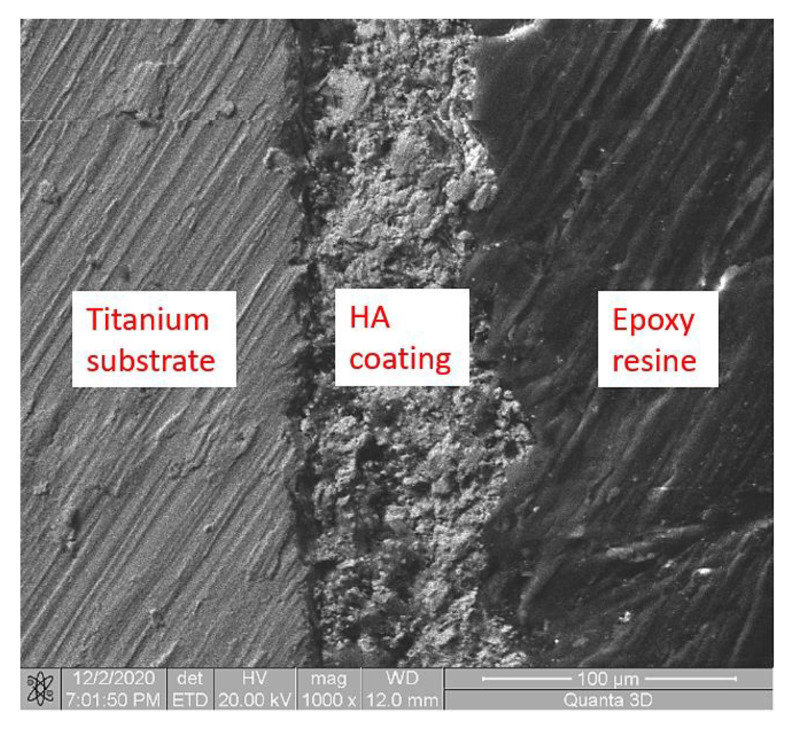
Representative aspects layer of HA on titanium alloys.

Figure 3 shows the hydroxyapatite layer deposited on the surface of titanium is uniform, with a thickness of the order of microns (Table 4) and contains crystals of nanometric dimensions as shown by natural bone. As shown in Table 4, the thickness of the samples decreased from 35 to 29 µm. Figure 4 shows a representative image of an HA layer on titanium alloys.

Hydroxyapatite coatings are often used for titanium implants in order to modify the surface properties. Most applications of HA coatings are for endosseous and subperiosteal dental implants and for orthopedic devices.

By coating the implant with a layer of hydroxyapatite, the implant benefits from biocompatibility, the ability to form chemical bonds with living bone and the mechanical properties of the titanium substrate. Due to the osteophilic surface of hydroxyapatite, the mechanical load acting on the implant will be transferred to the skeletal bone helping to combat bone atrophy [35,36,37].

The bone filling will form a skeleton and will facilitate the rapid filling of the pores by growing natural bone tissue. Hydroxyapatite as a filler is an alternative to bone grafts, becoming part of the bone structure and reducing the time required to heal diseased tissue.

Research in bone engineering shows that the structural properties of hydroxyapatite give it a better degree of resorbability and better osteoconductivity than dense hydroxyapatite. In addition, researchers recommend it as a good bone substitute for orthopedic implant surgery [38,39].

**Figure 5:**
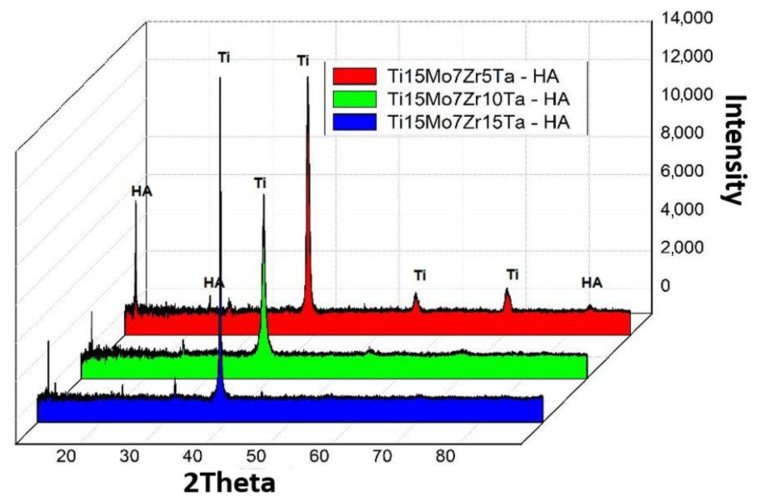
X-ray diffraction (XRD) graph of the coated alloys.

Figure 5 presents the X-Ray Diffraction (XRD) patterns of the coated alloys. It can be observed that the highest peaks observed in all the samples belonged to substrate metals S1, S2 and S3. This is because XRD beams can penetrate the thin coatings of hydroxyapatite and are diffracted from the underlying metals.

Figure 5 shows the result of X-ray diffraction obtained on the substrate samples of TMZT and were determined by some characteristic peaks of the substrate, which show the phases α and β, at an angle of 2θ: 35.40; 38.50; 39.45; 40.45; 53.30; 57.00; 63.55; 71.00; 74.90; 76.80; 78.10 with d (nm), respectively: 0.2536; 0.2338; 0.2284; 0.2230; 0.1719; 0.1614; 0.1464; 0.1328; 0.1268; 0.1241 and 0.1244. The β phase is clearly characterized by (110) and (200) and by the characteristic distortion reflection of the above-mentioned structure 0.2284 and 0.1614 nm respectively according to [40,41,42].

HA has a hexagonal crystal structure with the main one’s diffraction peaks at 2θ = 15.9; 29.0; 31.8; 32.2; 32.9; 34.0; 39.8; 46.7; 49.5; 50.5 and 83.1 corresponding to the crystal orientation planes (002), (210), (211), (112), (300), (202), (310), (222), (213), (321) and (004), as shown in Figure 5. The figure shows that the XRD model of the HA target is in accordance with the international standard, JCPDS 9-0432 [43].

Alloys show a crystalline structure HA with calcium phosphate (TCP) and Ca_3_(PO_4_)_3_ (PO4)_2_ that is formed during the sintering process.

### 3.2. Micro-Indentation Analysis

**Figure 6:**
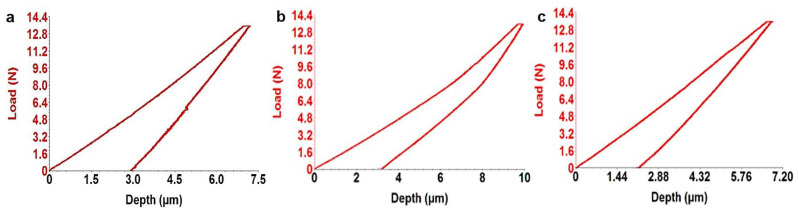
Micro-indentation graphic: (**a**) S1-HA, (**b**) S2-HA, (**c**) S3-HA.

Table 5 presents the micro-indentation analysis of coated samples’ results. Results are provided by the indentation test with the help of the VIEWER program which records them with the UMT 2 software (CETR, Campbell, CA, USA). The Young modulus obtained shows values between 55.35 ± 0.3 and 56.45.27 ± 0.3. This type of hydroxyapatite coating reduces the Young modulus of the Ti alloy (103–120 GPa) by 50% and makes them approach close to the cortical bone value (10–32 GPa). Figure 6 shows the response of the alloys during an indentation test, the force–displacement curve.

The more the coefficient of elasticity of the implant resembles the biological contingent tissue, the smaller the movement relative to the tissue-implant interface. Cortical bone is at least five times more flexible than titanium. As the size of the tension increases, the difference between the relative stiffness of the bone and the titanium increases. As the size of the tension decreases, the difference in relative stiffness between bone and titanium becomes much smaller. In this aspect, titanium alloys for orthopedic applications must have a modulus of elasticity as small as possible for long-term success [44].

### 3.3. Contact Angle Analysis

Biological hydroxyapatite is carbonated: half of the ${\mathrm{CO}}_{3}^{2-}$ groups are absorbed at the surface of the crystals, the other half being incorporated into the structure at positions XO_4_ and HO^−^. Biological hydroxyapatite contains ${\mathrm{HPO}}_{3}^{2-}$ hydrogen phosphate ions which justifies a Ca/P ratio lower than the theoretical value of 1.667. The presence of these ionic impurities also causes a distortion of the crystallographic cell of HA.

Biological hydroxyapatite also has important adsorption properties on its surface, properties which are determined by the presence of HO^−^ and ${\mathrm{PO}}_{3}^{2-}$ ions on the surface. In particular, water can bind very strongly to the HA surface. In addition, many organic molecules are absorbed at the surface of biological HA, playing an important role in controlling its growth.

**Figure 7:**
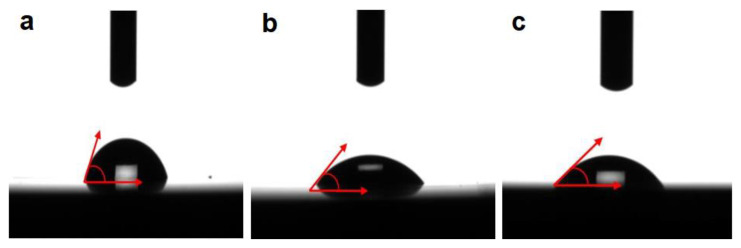
Images of the water drop on the surface of the alloys: (**a**) S1-HA, (**b**) S2-HA, (**c**) S3-HA.

For each experimental alloy, 10 measurements of the contact angle (θ) were performed, and the value presented is the average of the measurements performed, with a maximum error of ±1°. The average value of the contact angle for each alloy is shown in Table 6 and plotted in Figure 7. The wettability of a surface can be measured by the sessile drop technique, a method used for the characterization of solid surface energies, and in some cases, aspects of liquid surface energies. If the liquid used for the analysis is water, we are talking about hydrophilic/hydrophobic properties; a contact angle of θc < 90° indicates a high level of watering and a good hydrophilicity of the solid surface, whereas a contact angle of θc > 90° corresponds to a low level of watering of the surface, indicating its hydrophobicity. Regarding the biological response to changes in biomedical surfaces wettability, many researchers suggest that a general stimulating effect on the water contact is lower than 90°; therefore, the fluid will spread over a large area on the surface, whereas wetting surfaces with water contact above 90° is considered unfavorable.

The results show that the Ti15Mo7Zr5Ta-HA alloy has the highest value (73.61°) and the Ti15Mo7Zr10Ta-HA alloy (45.64°) has the lowest with a more pronounced hydrophilic character. All investigated alloys show a high adhesion of cells to the surface of the alloys, having a hydrophilic character with a contact angle of less than 90°.

The values of the TMZT-HA alloys obtained from the surface analysis by wetting certify that the developed titanium alloys have a good interaction with living tissues. Thus, we have the certainty of a good cellular adhesion, which could allow the “in vitro” analysis to determine a good biocompatibility in order to use the developed alloys in the human body.

## 4. Conclusions

The present study evaluated the coating of HA/tricalcium phosphate on titanium surfaces using the biomimetic method with positive results. The coatings on the substrates of Ti15Mo7Zr5Ta, Ti15Mo7Zr10Ta, and Ti15Mo7Zr15Ta showed a uniform morphological aspect and contained crystals of nanometric dimensions as shown by natural bone.

The biomimetic deposition of the hydroxyapatite layer on the metallic surfaces was achieved in a simulated physiological solution, and in a simulated body fluid (SBF) solution. The use of the biomimetic method induces the formation of a suitable layer of bone-like apatite in a very short time. Moreover, this technique of obtaining the coatings has the aim of increasing the bioactivity and bacteriostatic properties of the implants.

Micro-indentation showed a very low Young modulus with values between 55.35 ± 0.3 GPa and 56.45.27 ± 0.3 GPa, close to the human bone.

The surface wettability analysis value of the TMZT-HA alloy proves that the developed titanium alloys are predisposed to be biocompatible, with the Ti15Mo7Zr10Ta-HA alloy having a more pronounced hydrophilic character. These preliminary results suggest a good interaction with living tissue.

The coating with hydroxyapatite of titanium-based alloys elaborated and studied in the paper offer very promising results for future medical applications due to the properties of ceramics.

## Figures and Tables

**Table 1:** Metallic load for the experimental substrate alloys.

Alloy		Element (g)				Batch Weight (g)	Ingot Weight (g)	Efficiency (%)
		Ti	Mo	Zr	Ta			
S1	Ti15Mo7Zr5Ta	51.20	10.52	5.00	3.62	70.34	70.33	99.99
S2	Ti15Mo7Zr10Ta	63.02	15.08	7.04	15.00	70.55	70.45	99.86
S3	Ti15Mo7Zr15Ta	44.11	10.70	5.07	10.61	70.49	70.20	99.59

**Table 2:** Elemental compositions of substrate alloys.

Sample		Ti (wt.%)	Mo (wt.%)	Zr (wt.%)	Ta (wt.%)
S1	Average	73.94	14.57	6.75	4.74
	Stdev	±0.20	±0.30	±0.10	±0.10
S2	Average	68.75	15.10	6.80	9.35
	Stdev	±0.20	±0.10	±0.10	±0.30
S3	Average	63.71	14.85	6.99	14.45
	Stdev	±0.50	±0.30	±0.2	±0.20

**Table 3:** The ionic concentration (Mmol/L) of the simulated body fluid (SBF) solution vs. human blood plasma.

Ion	Na^+^	Ca^2+^	Mg^2+^	K^+^	Cl^−^	(HPO_4_)^2−^	(SO_4_)^2−^	(HCO_3_)^−^
SBF	142.0	2.5	1.5	5.0	147.8	1.0	0.5	4.2
Blood plasma	142.0	2.5	1.5	5.0	103.0	1.0	0.5	27.0

**Table 4:** The thickness of the deposited layers.

Sample	Layer Thickness (µm)
S1-HA	35 ± 3
S2-HA	29 ± 4
S3-HA	31 ± 2

**Table 5:** Micro-indentation results *.

Sample	Loading Deformation (N)	Release Deformation (μm)	Young Modulus (GPa)	Stiffness (N/μm)	Specimen Poisson Ration
S1-HA	13.35 ± 0.4	12.75 ± 0.3	55.35 ± 0.3	6.35 ± 0.1	0.27
S2-HA	13.25 ± 0.5	12.10 ± 0.1	56.25 ± 0.2	6.25 ± 0.2	0.27
S3-HA	13.75 ± 0.4	12.35 ± 0.2	56.45 ± 0.3	5.75 ± 0.2	0.27

* Five determinations were carried out in different areas of the samples with dimensions of 10 mm × 10 mm × 5 mm.

**Table 6:** Water contact angle values on the surface of alloys studied.

Alloy	S1-HA	S2-HA	S3-HA
Liquid used	Water		
Contact angle (degrees)	73.61	45.64	57.93

## Data Availability

Not applicable.
